# Hair Mercury Concentrations and Fish Consumption Patterns in Florida Residents

**DOI:** 10.3390/ijerph110706709

**Published:** 2014-06-26

**Authors:** Adam M. Schaefer, Emily L. Jensen, Gregory D. Bossart, John S. Reif

**Affiliations:** 1 Harbor Branch Oceanographic Institution at Florida Atlantic University, 5600 U.S. 1 North, Fort Pierce, FL 34946, USA; 2 Department of Environmental and Radiological Health Sciences, Colorado State University 1681 Campus, Fort Collins, CO 80523, USA; E-Mails: Emily.Jensen@childrenscolorado.org (E.L.J.); John.Reif@colostate.edu (J.S.R.); 3 Georgia Aquarium, 225 Baker Street NW, Atlanta, GA 30313, USA; E-Mail: gbossart@georgiaaquarium.org

**Keywords:** mercury, dietary exposure, fish consumption, sentinel species, Florida, bottlenose dolphins

## Abstract

Mercury exposure through the consumption of fish and shellfish represents a significant public health concern in the United States. Recent research has demonstrated higher seafood consumption and subsequent increased risk of methylmercury exposure among subpopulations living in coastal areas. The identification of high concentrations of total mercury in blood and skin among resident Atlantic bottlenose dolphins (*Tursiops truncatus*) in the Indian River Lagoon (IRL), a coastal estuary in Florida, alerted us to a potential public health hazard in the contiguous human population. Therefore, we analyzed hair mercury concentrations of residents living along the IRL and ascertained their sources and patterns of seafood consumption. The total mean mercury concentration for 135 residents was 1.53 ± 1.89 µg/g. The concentration of hair mercury among males (2.02 ± 2.38 µg/g) was significantly higher than that for females (0.96 ± 0.74 µg/g) (*p* < 0.01). Log transformed hair mercury concentration was significantly associated with the frequency of total seafood consumption (*p* < 0.01). Individuals who reported consuming seafood once a day or more were 3.71 (95% CI 0.84–16.38) times more likely to have a total hair mercury concentration over 1.0 µg/g, which corresponds approximately to the U.S. EPA reference dose, compared to those who consumed seafood once a week or less. Hair mercury concentration was also significantly higher among individuals who obtained all or most of their seafood from local recreational sources (*p* < 0.01). The elevated human mercury concentrations mirror the elevated concentrations observed in resident dolphins in the same geographical region. The current study is one of the first to apply the concept of a sentinel animal to a contiguous human population.

## 1. Introduction

Mercury is a global environmental pollutant with important adverse health effects, particularly on neurodevelopment in the fetus. This relationship was first brought to light in the well known episode at Minamata Bay, Japan where pregnant women were exposed to high concentrations of methylmercury (MeHg) by eating contaminated seafood [1]. Subsequent long term cohort studies of residents of the Faroe Islands [2] confirmed and extended these findings to indicate that prenatal exposure to MeHg is associated with cognitive and psychomotor impairment in children, although some controversy persists due to conflicting results from other populations [3]. Prenatal exposure to mercury can result in multiple neurodevelopmental effects including deficits in memory, language, attention and fine motor skills [4,5,6]. Current evidence suggests that adult exposure to MeHg may also have subtle effects on the nervous, immune and cardiovascular systems, although these associations are weaker and less consistent than those associated with pre-natal exposure [7]. 

Human exposure to MeHg is primarily from the consumption of fish and shellfish [5]. Several studies have demonstrated an association between dietary intake of fish and the accumulation of mercury in the body [8,9,10]. Coastal subpopulations often consume more fish than the general population [11]. This increase is likely driven by regional access to fresh seafood [12]. As a result, coastal human populations typically have higher concentrations of mercury in their tissues compared to inland populations [13,14,15]. In Florida, the average adult consumes approximately 46 g per day of seafood, considerably higher than the estimated 4.5 g per day for the general population in the United States [13]. In a survey of sport fish consumption among women of childbearing age from twelve states, women in Florida consumed more per year than those from other states [14]. Further, environmental concentrations of mercury are generally higher than those found in other states [16]. Therefore, there is a need for regional estimates of mercury exposure among individuals who consume seafood at a higher frequency than the general population of the US. 

A variety of industrial processes release mercury into the atmosphere which is subsequently deposited in aquatic environments. Inorganic mercury in the aquatic environment is converted to MeHg through bacterial action and bioaccumulates through trophic levels of the food web [17]. As a result, apex predators accumulate the highest tissue concentrations of mercury in the ecosystem. We became aware of a potential public health risk to humans along the Florida coastline through studies of mercury accumulation in blood and skin samples of Atlantic bottlenose dolphins (*Tursiops truncatus*) inhabiting the Indian River Lagoon, FL (IRL). These analyses measured total mercury (THg) concentrations of 658 ± 519 μg/L wet weight in blood [18] and 7.0 ± 5.9 μg/g dry weight in skin [19]. Approximately 73 percent of THg in dolphins was MeHg [19]. Concentrations in blood and skin were more than four times higher than those found in bottlenose dolphins sampled in Charleston Harbor, SC [18,19]. Elevated mercury concentrations were associated with perturbation of multiple hepatic, renal, endocrine, and hematological parameters suggesting deleterious health effects in adult dolphins [20]. High concentrations of mercury in skin have also been found in dolphins from Florida’s west coast [21] and the Mediterranean Sea [22].

As an apex predator, Atlantic bottlenose dolphins in the IRL have a long life span, bioaccumulate anthropogenic contaminants and have defined home ranges making them a valuable sentinel species [23]. The high mercury concentrations found in IRL dolphins may reflect environmental differences in mercury contamination that include deposition, biogeochemistry and trophic transfer [24]. Similarly, these factors impact the concentration of mercury found in fish species that local human populations may be consuming. Therefore, the main objective of this study was to examine exposure to mercury among coastal residents living near the IRL. The secondary objective was to examine associations between the frequency, species and sources of seafood consumed by residents and their hair mercury concentration. 

## 2. Methods

### 2.1. Study Population

**Figure 1:**
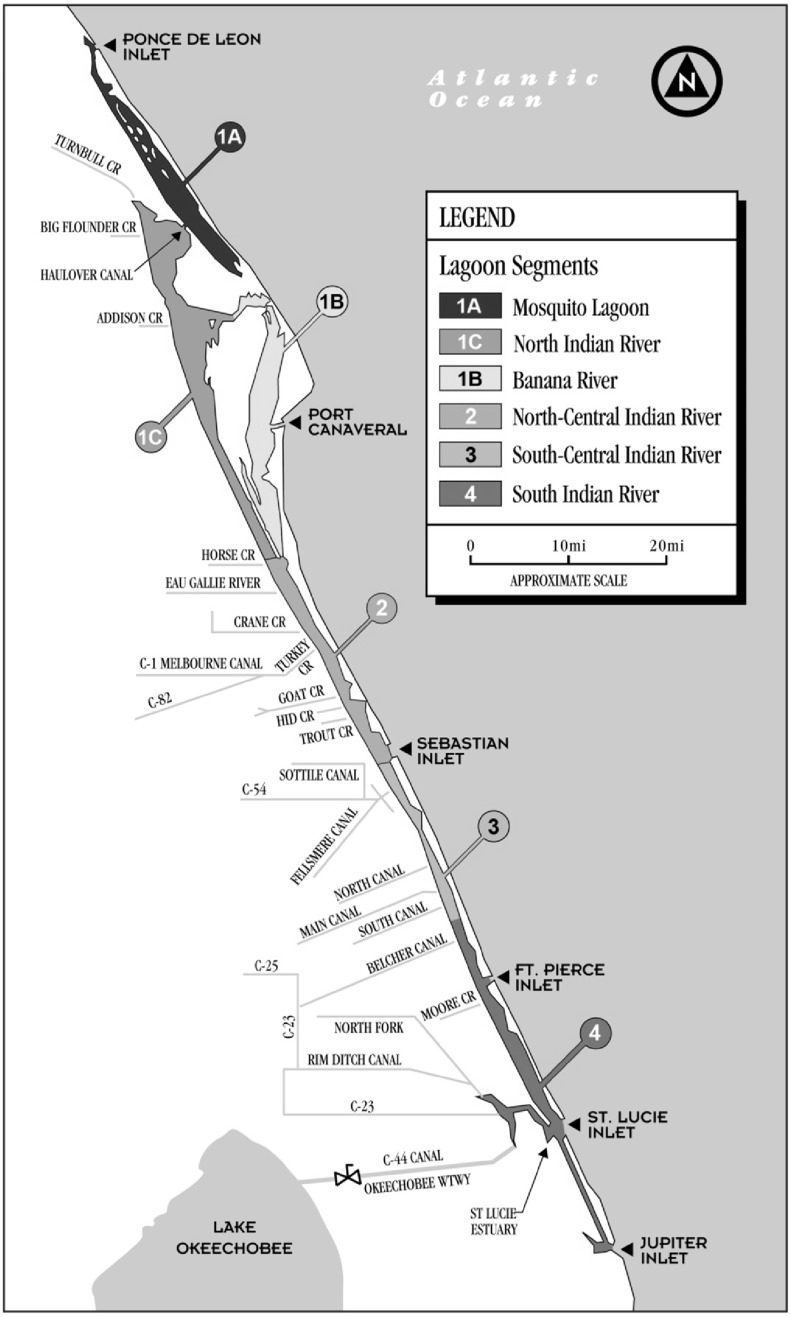
Map of the Indian River Lagoon with bordering counties and spatial segments of the estuary.

Individuals living along the Indian River Lagoon, Florida were recruited during June and July of 2011 and 2012. Residents of Volusia, Brevard, Indian River, St. Lucie, Martin and Palm Beach counties were included (Figure 1). Participants were recruited from public meeting events, seafood markets, popular fishing sites and other outdoor locales, such as bait shops in a convenience based sampling scheme. Eligibility criteria included being 18 years of age or older and having the ability to understand and consent to participation. The recruitment process, informed consent and study protocol were approved by the Institutional Review Board of Florida Atlantic University. 

### 2.2. Questionnaire Design and Administration

The questionnaire was modified from that used by Lincoln *et al.* [11] with questions added to assess the sources of seafood consumed, specific local fish species, zip code of residence and additional demographic variables specific to Florida residents. All participants were questioned in person by trained interviewers prior to hair sampling at the site of recruitment. The recall period (3 months) was chosen to approximate the one to three months exposure period represented by the hair biomarker. Hair has been validated as a reliable and non- invasive biomarker of MeHg intake in human populations [25,26,27]. Participants were asked to recall the frequency of their total fish and shellfish consumption from the following choices: never, once a month or less, once a week, three times a week, or once or more a day. Frequency of consumption of individual fish species was assessed using the same categories for 12 common sportfish. Participants were asked to provide information for consumption of sportfish not specifically mentioned in the questionnaire. Participants were also asked the source of the seafood they consumed to compare individuals who ate primarily recreationally caught seafood with those who obtained seafood commercially from stores or restaurants. 

### 2.3. Hair Sample Collection and Mercury Analysis

After completing the questionnaire, all participants were asked to provide a hair sample for analysis of total mercury concentration. A bundle of hair approximately 3 mm in diameter was cut from the occipital region of the head using stainless steel scissors and placed in a plastic collection bag. Two centimeters of the proximal end of each sample were sent to Quicksilver Scientific, Lafayette, CO for analysis of total mercury concentration. Thermal decomposition, amalgamation, and atomic absorption spectrophotometry were used following EPA method 7473 on a Milestone Direct Mercury Analyzer (Milestone Inc., Shelton, CT, USA). Total hair mercury concentration was measured and reported as µg/g. 

### 2.4. Statistical Analysis

Descriptive statistics for the study population including age, gender, race and education were calculated. Body mass index (BMI) was calculated from participants’ weight and height. Participants’ zip code was used to assign county of residence.

The distribution of total hair mercury concentrations was described by calculating mean, standard deviation and percentile (50th, 75th and 90th) for the entire population and for demographic and specific response variables. The normally distributed transformed variable was used in all subsequent analyses. Differences across demographic variables, fish consumption responses and two categories of species specific fish consumption (<once a week and ≥ a week) were compared initially using an independent sample *t*-test and an analysis of variance when appropriate. Data were pooled and stratified by gender for analysis in order to examine potential effect modification. The consumption categories of seafood obtained from the IRL were combined (three times a week or more *vs.* once a week or less) due to sample size limitations.

The influence of participant’s fish consumption on log-hair mercury concentration was further explored using multivariable linear regression. A forward addition model building approach to control for potential confounding factors was used and variables for the final model were included if they contributed >10% to the adjusted R^2^ value. The final linear model included total seafood consumption, gender, education and the source of fish and shellfish consumed by the participant. Separate analyses were also done by gender using the variables described above. In a second approach, hair mercury concentration was categorized as <1.0 µg/g and ≥1.0 µg/g in order to calculate odds ratios (OR) with their 95% confidence intervals (CI) using logistic regression. This value corresponds approximately to the U.S. EPA’s reference dose of 0.1 μg/kg/day [28]. Univariate analyses were conducted using categorized strata of total seafood consumption (once a day or more, three times per week, once a week or less), and sources of fish and shellfish consumed (all or most from recreational sources versus half or more obtained commercially from a store or restaurant). Categories were collapsed as required based on sample size limitations. All analysis was conducted using IBM SPSS statistics 20 for windows (IBM Corp. 2011, Armonk, NY, USA).

## 3. Results

### 3.1. Study Population Demographics and Fish Consumption Patterns

Seafood consumption information and hair samples were provided by a total of 135 people; 73 men and 62 women. Participants were predominantly white/Caucasian (95%) with a mean age of 54 years (18–90) and a mean BMI of 25.7 (kg/m^2^). They were relatively well-educated. Fifty-six percent had attained a college degree or had post-graduate education, while 21 percent had only a high school education or less (Table 1). 

Participants reported overall seafood consumption, including fish and shellfish over the past 3 months; 7% reported eating seafood once or more per day, 49% three times a week, 37% once a week and 7% reported eating seafood once per month or less. In the gender-stratified analysis, 31% of males reported consuming only recreationally caught fish and 16% reported that most of the fish they consumed was recreationally caught. In contrast, only 9% of females reported only consuming recreationally caught fish and 8% reported that most of the fish they consumed was recreationally caught. Additionally, 79% of females reported that all of the shellfish they consumed was from a store or restaurant compared to 50% of males.

**Table 1:** Total hair mercury concentration (µg/g) by demographic and fish consumption variables, Florida 2011–2012.

Participant Group						Percentile			
	*N*	*%*	Mean ± SD	Range	Median	75th	90th	95th	*p*-value ^1^
All	135		1.53 ± 1.89	0.04–16.18	1.01	1.86	3.16	5.01	
*Sex*									<0.01
Male	73	54%	2.02 ± 2.38	0.07–16.18	1.17	2.81	4.74	6.06	
Female	62	46%	0.96 ± 0.74	0.04–3.26	0.74	1.38	1.98	2.60	
*Age*									0.24
18 – 40	29	22%	1.07 ± 0.73	0.04–2.72	0.96	1.62	2.34	2.57	
41 – 60	53	39%	1.75 ± 1.83	0.19–8.09	1.01	2.48	5.06	6.06	
61+	53	39%	1.57 ± 2.33	0.07–16.18	1.07	1.84	3.21	4.30	
*Race/Ethnicity*									0.69
Caucasian	129	95%	1.56 ± 1.93	0.04–16.18	1.01	1.86	3.17	5.05	
Other	6	5%	1.08 ± 0.90	0.31–2.58	0.77	1.90	-	-	
*Education*									0.31
High school or less	28	21%	1.78 ± 1.96	0.07–8.09	1.02	2.92	5.02	6.88	
Some college	31	23%	1.09 ± 1.37	0.04–7.58	0.75	1.45	2.44	4.61	
Graduated college or more	76	56%	1.63 ± 2.04	0.08–16.18	1.12	2.17	3.15	4.39	
*BMI (kg/m^2^)*									0.47
<24.9	54	40%	1.26 ± 1.24	0.04–7.58	0.86	1.50	2.68	3.32	
25–29.9	55	42%	1.99 ± 2.57	0.07–16.18	1.20	2.58	5.03	5.94	
>30	25	18%	1.16 ± 0.94	0.14–3.17	0.96	1.64	2.99	3.13	
*County of Residence*									0.03
Brevard	33	25%	1.88 ± 1.79	0.08–8.09	1.17	2.79	4.82	6.14	
Indian river	18	14%	0.97 ± 0.79	0.07–2.94	0.78	1.50	2.39	–	
St. Lucie	22	17%	1.07 ± 0.74	0.14–3.17	0.82	1.46	2.26	3.05	
Martin	38	28%	2.08 ± 2.81	0.18–16.18	1.17	2.65	4.33	8.01	
Palm beach	5	4%	1.34 ± 0.74	0.62–2.22	1.09	2.13	–	–	
Other	16	12%	0.95 ± 1.38	0.04–5.41	0.50	1.02	3.70	–	
*Total Seafood Consumption*									<0.01
Once or more per day	9	7%	2.14 ± 1.86	0.04–5.31	2.96	3.21	–	–	
Three times per week	66	49%	1.95 ± 2.32	0.08–16.18	1.20	2.39	4.30	5.30	
Once per week	50	37%	1.08 ± 1.16	0.09–7.58	0.73	1.41	2.02	2.84	
Once per month or less	10	7%	0.49 ± 0.29	0.18–0.91	0.39	0.79	0.90	–	
*IRL Seafood Consumption*									0.11
Three times per week or more	8	6%	2.01 ± 1.47	0.79–4.98	1.19	3.02	–	–	
Once per week	17	13%	1.71 ± 1.41	0.26–5.41	1.14	3.07	3.69	–	
Once per month or less	110	81%	1.47 ± 1.99	0.04–16.18	0.89	1.73	2.96	4.71	
*Fish Sources*									<0.01
All recreational	28	21%	2.53 ± 3.20	0.26–16.18	1.21	3.14	5.36	12.31	
Most recreational	17	13%	2.46 ± 2.24	0.07–8.09	1.62	3.76	5.95	–	
Half recreational	13	10%	1.65 ± 1.06	0.49–3.63	1.15	2.71	3.44	–	
Most commercial	24	18%	1.20 ± 0.71	0.18–2.58	1.14	1.73	2.31	2.52	
All commercial	52	38%	0.85 ± 0.73	0.04–2.96	0.60	1.11	1.85	2.72	
*Shellfish Sources*									<0.01
All recreational	14	10%	3.37 ± 4.50	0.19–16.18	1.20	5.63	12.14	–	
Most recreational	10	7%	2.54 ± 1.83	0.55–5.41	1.84	4.47	5.38	–	
Half recreational	5	4%	2.77 ± 1.15	1.15–4.38	2.72	3.68	–	–	
Most commercial	19	15%	1.10 ± 0.82	0.20–3.63	0.85	1.41	2.28	–	
All commercial	85	64%	1.12 ± 1.00	0.04–5.31	0.75	1.60	2.74	3.08	

^1^ *p*-value from independent sample *t*-test or ANOVA using log Hg.

### 3.2. Hair Mercury Concentration

Total hair mercury concentrations in participants ranged from 0.04 to 16.18 µg/g with a mean of 1.5 µg/g and median of 1.0 µg/g. There were no statistically significant relationships between mercury concentrations and age, education, BMI, or ethnicity in the descriptive analysis. However, log total hair mercury concentration was significantly different across gender (*p* < 0.01), total seafood consumption (*p* < 0.01), county of residence (*p* = 0.03), and fish and shellfish sources (*p* < 0.01) (Table 1). Male participants had a higher mean mercury concentration compared to female participants 2.02 ± 2.38 and 0.96 ± 0.74 µg/g respectively (*p* < 0.01). Fulltime residents of Martin county had the highest mean hair concentration (2.08 ± 2.81 µg/g) followed by Brevard (1.88 ± 1.79 µg/g) and Palm Beach counties (1.34 ± 0.74 µg/g) (Table 1). Total hair mercury concentration was significantly associated with total seafood consumption (*p* < 0.01). Concentrations were highest among individuals who reported consuming seafood daily (2.14 ± 1.86 µg/g) and decreased stepwise with each descending category of total consumption (Table 1). The same pattern was observed for frequency of consumption of recreationally caught IRL fish; however, the relationship was not statistically significant (*p* = 0.11). Individuals who consumed seafood from the IRL three times a week or more had the highest hair mercury concentration of 2.01 ± 1.47 µg/g while those who reported eating IRL seafood once a month or less had a mean concentration of 1.47 ± 1.99 µg/g. Hair mercury concentration was also significantly different by source (*p* < 0.01) with the highest concentration among participants who reported that all fish and shellfish consumed were from local recreational sources, 2.53 ± 3.20 µg/g and 3.37 ± 4.50 µg/g, respectively (Table 1). 

Log transformed total hair mercury concentration was compared for the most commonly reported species consumed. Mean mercury concentrations were significantly higher among individuals who consumed snapper, sea trout, cobia, and grouper once a week or more compared to those who ate these fish less than once a week (Table 2).

A multivariable linear regression model was developed using a forward addition model building approach (Table 3). Statistically significant associations were found for education and source of seafood after adjusting for total seafood consumption and gender. Participants who had graduated from college had significantly higher log Hg concentrations than those who had only a high school education. Participants who obtained most or all of their fish from recreational sources had higher log Hg concentrations than those that consumed fish only from stores or restaurants. 

Separate linear regression models were run for males and females using the same variables (Table 4). The only significant differences were found among males. A significant association with log Hg concentrations was found among men who consumed all of their fish from recreational sources compared to men consuming fish only from stores or restaurants. 

Univariate logistic regression was used to examine the associations between gender, total seafood consumption and sources of seafood and a hair mercury concentration at or above 1.0 µg/g (Table 5). The results were in general agreement with those from the linear regression and ANOVA analyses with respect to total seafood consumption and sources of seafood. Males had a statistically significant increased risk (OR = 2.12, 95% CI 1.06–4.23) of having a mercury concentration at or above 1.0 µg/g compared to females. 

**Table 2:** Mean total hair mercury concentration (µg/g) by frequency of fish and shellfish consumption and species, Florida 2011–2012.

Species	<Once a Week	≥Once a Week	*p*-value^ 1^
Canned Tuna	1.51 (1.97)	1.63 (1.60)	0.19
Tuna Fillet or Steak^ 2^	1.51 (1.95)	1.80 (1.05)	0.12
Salmon	1.52 (2.02)	1.56 (1.48)	0.39
Snapper ^2^	1.38 (1.88)	2.71 (1.55)	<0.01
Mullet ^2^	1.49 (1.89)	2.68 (1.78)	0.06
Sea Trout^ 2^	1.49 (1.94)	2.21 (0.87)	0.02
Cobia	1.29 (1.23)	4.99 (4.68)	<0.01
Shark ^2^	1.54 (1.90)	-	-
Swordfish^ 2^	1.51 (1.90)	2.80 (0.24)	0.13
Grouper^ 2^	1.43 (1.93)	2.16 (1.55)	0.02
Shrimp and Crab	1.72 (2.27)	1.34 (1.39)	0.19
Shellfish other^ 2^	1.40 (1.42)	1.94 (2.86)	0.12
Sushi	1.53 (1.97)	1.56 (1.28)	0.30
Tilapia	1.59 (1.95)	0.83 (0.57)	0.09
Mahi^ 2^	1.52 (1.99)	1.63 (1.25)	0.18
Flounder^ 2^	1.53 (1.90)	1.80 (1.64)	0.60
Pompano	1.53 (1.90)	2.16 (1.40)	0.32
Redfish^ 2^	1.53 (1.90)	2.62 -	-
Cod	1.54 (1.93)	1.33 (0.62)	0.64
Sheepshead^ 2^	1.54 (1.90)	1.17 -	-
All Other Species	1.44 (1.42)	2.84 (5.15)	0.91

^1^ Independent sample *t*-test using log total mercury; ^2 ^Indicates species found in the region.

**Table 3:** Multivariable linear regression analysis of demographic variables, fish consumption frequency and sources of fish consumed on log total hair mercury concentration.

Variable	Β-Coefficient	95% Confidence Interval	*p*-value^ 1^
*Gender*			
Females	Ref	-	-
Males	0.11	−0.05–0.27	0.17
*Education*			
High school or less	Ref	-	-
Some college	0.07	−0.29–0.14	0.26
Graduated college or more	0.15	0.03–0.30	0.05
*Total Seafood Consumption*		-	-
≤1x/month	Ref	-	-
1x/week	0.19	0.11–0.49	0.20
≥3x/week	0.26	0.04–0.56	0.08
*Fish Sources*			
All commercial	Ref	-	-
Most commercial	0.10	−0.11–0.32	0.34
Half recreational	0.22	−0.05–0.49	0.11
Most recreational	0.32	0.08–0.56	0.01
All recreational	0.32	0.11–0.53	<0.01

^1^ final model adjusted for gender, education, total seafood consumption, and source of fish.

**Table 4:** Gender stratified multivariable linear regression of demographic variables, fish consumption frequency and sources of fish consumed on log total hair mercury concentration.

Variable	β-Coefficient	95% Confidence Interval	*p*-value
***Male Participants***			
*Education*			
Less than college	Ref	-	-
Graduated from college or more	0.10	0.11–0.32	0.34
*Total Seafood Consumption*			
≤1x/month	Ref	-	-
1x/week	−0.09	−0.74–0.56	0.78
≥3x/week	0.09	−0.56–0.74	0.78
*Fish Sources*			
All commercial	Ref	-	-
Most commercial	0.28	0.06–0.62	0.10
Half recreational	0.39	0.04–0.74	0.03
Most recreational	0.47	0.12–0.81	0.01
All recreational	0.50	0.21–0.80	<0.01
***Female Participants***			
*Education*			
Less than college	Ref	-	-
Graduated college or more	0.19	0.03–0.40	0.08
*Total Seafood Consumption*			
≤1x/month	Ref	-	-
1x/week	0.26	−0.08–0.60	0.13
≥3x/week	0.23	−0.13–0.58	0.20
*Fish Sources*			
All commercial	Ref	-	-
Most commercial	0.05	−0.20–0.30	0.70
Half recreational	0.19	−0.33–0.72	0.46
Most recreational	0.15	0.19–0.50	0.38
All recreational	0.23	−0.11–0.57	0.18

**Table 5:** Risk estimates (OR and 95% CI) for hair mercury concentration <1.0 *vs.* ≥1.0 µg/g for gender, total seafood consumption and sources of seafood.

Risk Factor	< 1 µg/g (n = 67)	≥ 1 µg/g (n = 68)	OR	95% CI	
				Lower	Upper
*Gender*					
Male	30	43	2.12	1.06	4.23
Female	37	25	1.00	-	-
*Total Seafood Consumption*					
Once a day or more	3	6	3.71	0.84	16.38
Three times per week	25	41	3.05	1.47	6.30
Once per week or less	39	21	1.00	-	-
*Fish Sources*					
All or > 50% recreationally	16	29	2.32	1.11	4.87
Half or more commercially	50	39	1.00	-	-
*Shellfish Sources*					
All or > 50% recreationally	7	17	2.98	1.14	7.75
Half or more commercially	60	49	1.00	-	-

Individuals who reported consuming seafood once a day or more were 3.71 (95% CI 0.84–16.38) times more likely to have a hair mercury concentration at or above 1.0 µg/g compared to those who consumed seafood once a week or less. Individuals who consumed seafood three times a week were 3.05 (95% CI 1.47–6.30) times more likely to have a mercury concentration at or above 1.0 µg/g compared to those who consumed seafood once a week or less. Sources of seafood were also statistically significant risk factors. Individuals who reported eating all or most of their fish from local recreational sources were 2.32 (95% CI 1.11–4.87) times more likely to have a mercury concentration at or above 1.0 µg/g compared to those who reported that half or more of their fish came from a store or restaurant. Similarly, individuals who reported eating all or most of their shellfish from recreational sources were 2.98 (95% CI 1.14–7.75) times more likely to have a mercury concentration at or above 1.0 µg/g compared to those who reported that half or more of their shellfish came from a store or restaurant.

## 4. Discussion

To the authors’ knowledge, this is the first attempt to assess hair mercury concentrations and seafood consumption among recreational anglers and residents living along the east coast of Florida. It is also the first to apply findings from bottlenose dolphins as a stimulus to explore the potential for similar risk among humans from the same geographical region. Study participants had a mean hair mercury concentration of 1.5 µg/g. The U.S. EPA exposure guideline, which equates approximately to a hair mercury concentration of 1 µg/g [28], was exceeded in 50% of the samples obtained from participants. The concentrations found in Florida women of all ages in this study (0.96 µg/g) were approximately five times higher than those from a randomly generated sample of US women of childbearing age (0.19 µg/g) from NHANES [29]. In addition, the concentrations of hair mercury in this study were higher than those reported from anglers and coastal resident populations in Canada (0.82 µg/g), Wisconsin (0.86 µg/g), Alabama (0.55 µg/g) and Louisiana (1.1 µg/g) [9,11,15,30].

Nearly 45 percent of study participants reported consuming any seafood once a week or less, a lower frequency than the FDA recommended 12 ounces (two average meals) per week of seafood containing low concentrations of mercury [31]. However, 56 percent of those surveyed reported eating fish and shellfish three times per week or more which is higher than the recommended frequency. Participants consumed a variety of fish species. Commercial market data showed that typical US consumers derive most of their methylmercury exposure from a narrow range of fish and shellfish types, most of which were imported, not locally caught [32]. At the national level, estimated Hg intake is associated with a few commonly consumed fish types with moderate to high concentrations of Hg, such as canned tuna (0.35 µg/g) and swordfish (0.98 µg/g) [32]. In the current study, individuals who consumed locally caught grouper, sea trout and snapper at least once a week had significantly higher hair mercury concentrations compared to those who consumed these species less than once a week in the past three months. 

Males had a significantly higher concentration of hair mercury and were 2.12 times more likely to have a total hair mercury concentration above 1µg/g compared to females. Similar differences in concentration by gender have been reported from Japan, Wisconsin and Louisiana [9,11,33]. A probable explanation for these results is that males tend to do more recreational fishing and consumption of their catch compared to females. Previous studies have demonstrated that the main sources of seafood for females tend to be shrimp and canned tuna from grocery stores and seafood markets [34]. The same pattern was seen in the current study. In addition, men commonly consume more fish per kg of bodyweight during a meal compared to females [9]. As a result, males who are frequent recreational anglers represent a highly exposed subpopulation along the Indian River Lagoon.

Age was not associated with increased mercury concentration. The lack of an association with age was also reported among women of childbearing age in Florida [35] and sportfish consumers in Canada [30]. However, other studies have found that hair mercury concentration was associated with increased age [12,14]. Native populations had the highest reported mercury concentrations in multiple studies compared to other ethnic groups [12,36,37]. However, potential differences in mercury concentration and fish consumption patterns by race and ethnicity could not be evaluated in this study since the sample was almost exclusively caucasian. 

Higher levels of educational attainment were associated with hair mercury concentration in current analyses for the total population but not in gender-stratified analyses. Education and high household income (>$75,000/year) were also associated with increased hair mercury concentration and shellfish consumption in a 12 state study of women and with awareness of state-issued consumption advisories for fish consumption [14]. Higher socioeconomic status may be linked to increased access to recreationally acquired sportfish and the ability to purchase relatively expensive foodstuffs. In addition, persons with higher levels of education may be more aware of the health benefits associated with fish consumption.

There was a statistically significant difference in hair mercury concentration by county of residence with residents of Martin county having the highest mean hair mercury concentration (2.08 ± 2.81 µg/g) followed by Brevard county (1.88 ± 1.79 µg/g). The differences could not be explained by differences in the proportion of males in these two counties. However, a higher proportion of participants from Martin County reported consuming seafood three times a week or more compared to other counties. Both counties border directly on the IRL and have higher mean income and proportion of residents with at least a high school education compared to the general population in Florida [38].

Several limitations of this study are important in interpreting the findings. Although identification of high concentrations of mercury in bottlenose dolphins led to the identification of high concentrations of mercury among coastal human residents, a quantitative comparison of the concentrations between species was not possible. Human exposure was assessed in hair and dolphin exposure in skin and blood. Second, although both dolphins and humans consumed fish from the same environment, the species of fish consumed differed. IRL dolphins consume a majority of their diet as striped mullet, silver perch and spot [39] whereas human exposure was driven by those who reported consuming cobia, snapper, grouper and sea trout. Neither the human nor the dolphin data permitted a direct examination of methylmercury concentration, the most toxic form of the element. Since the participants were obtained through opportunistic rather than random sampling, the results cannot be generalized. Selection bias may well have occurred if the volunteer participants recruited through opportunistic sampling had higher concentrations of mercury in hair than non-participants. However, this sample of Florida residents contained substantial numbers of persons who obtained most or all of their seafood from commercial sources, permitting comparison of mercury exposure across frequency of seafood consumption and source of fish and shellfish. In addition, information bias resulting in under- or over- estimation of seafood consumption during the prior three months could have occurred. Since participants had no knowledge of their hair mercury concentrations at the time of interview, this form of bias would be expected to be non-differential with respect to their hair mercury concentration and lead to underestimation of risk.

In summary, these data provide insight into regional mercury exposure in Florida where access to and consumption of seafood is higher. In addition, mercury contamination in generally higher in Florida compared to all other states [16]. Since our sample population is highly exposed to mercury, targeted education and local advisories should be designed to reduce regionally specific exposure pathways. Future local consumption advisories may include several of the species identified in this study, particularly for pregnant women. In contrast, there are well-recognized benefits of fish consumption for pregnant women and the general population. Positive effects on early cognitive development in children and a reduced risk of cardiovascular disease in adults have been attributed to the n-3 polyunsaturated fatty acids found to varying degrees in commonly consumed fish species [40]. The challenge for public health is to find and recommend the balance between the positive and negative effects of fish and shellfish consumption.

## 5. Conclusions

The similarity between high concentrations of mercury in human hair and previously reported concentrations in dolphin skin from eastern Florida exemplifies the concept of a sentinel animal for identification of a public health hazard. The findings of high concentrations of mercury in hair among coastal residents in eastern Florida associated with consumption of locally caught seafood and specific species of fish should be used to develop interventions to reduce exposure among high risk groups, particularly pregnant women. 
